# A Novel Two-Component System Involved in Secretion Stress Response in *Streptomyces lividans*

**DOI:** 10.1371/journal.pone.0048987

**Published:** 2012-11-14

**Authors:** Sonia Gullón, Rebeca L. Vicente, Rafael P. Mellado

**Affiliations:** Centro Nacional de Biotecnología (CNB-CSIC), Madrid, Spain; Centre National de la Recherche Scientifique, Aix-Marseille Université, France

## Abstract

**Background:**

Misfolded proteins accumulating outside the bacterial cytoplasmic membrane can interfere with the secretory machinery, hence the existence of quality factors to eliminate these misfolded proteins is of capital importance in bacteria that are efficient producers of secretory proteins. These bacteria normally use a specific two-component system to respond to the stress produced by the accumulation of the misfolded proteins, by activating the expression of HtrA-like proteases to specifically eliminate the incorrectly folded proteins.

**Methodology/Principal Findings:**

Overproduction of alpha-amylase in *S. lividans* causing secretion stress permitted the identification of a two-component system (SCO4156-SCO4155) that regulates three HtrA-like proteases which appear to be involved in secretion stress response. Mutants in each of the genes forming part of the two-genes operon that encodes the sensor and regulator protein components accumulated misfolded proteins outside the cell, strongly suggesting the involvement of this two-component system in the *S. lividans* secretion stress response.

**Conclusions/Significance:**

To our knowledge this is the first time that a specific secretion stress response two-component system is found to control the expression of three HtrA-like protease genes in *S. lividans*, a bacterium that has been repeatedly used as a host for the synthesis of homologous and heterologous secretory proteins of industrial application.

## Introduction

As in most bacteria, the Sec pathway is the main route for secretory proteins exported across the cytoplasmic membrane in *Streptomyces* bacteria [1], [2]. Upon exit from the membrane translocase complex, the proteins must acquire a properly folded conformation to gain full activity. Heat stress and oversynthesis of specific secretory proteins can result in the accumulation of misfolded proteins outside the cytoplasm [3]. The accumulation of misfolded proteins produces cellular stress responses leading to refolding or degradation of the abnormally folded, non-functional proteins [4].

This secretion stress is sensed in the Gram-negative *Escherichia coli* bacterium by the CpxRA two-component system and the extra-cytoplasmic sigma factor σ^E^ [5], [6]. CpxA acts as a sensor histidine kinase that upon stimuli undergoes autophosphorylation. The phosphorilated sensor kinase transfers this sensed information by activating the cognate response regulator CpxR via phosphorilation. Phosphorilated CpxR activates the HtrA serine protease, which could act as a chaperone or protease, depending on the temperature; the protease and chaperone activities of HtrA respectively eliminate or refold unfolded proteins in the bacterial periplasm [7].

In the Gram-positive *Bacillus subtilis*, bacterium secretion stress is sensed at least by the CssRS two-component system (a homologue of the *E. coli* CpxRA) [8] where CssS is the sensor histidine kinase and CssR the response regulator. The activation of CssRS induces the synthesis of two HtrA-like proteases, HtrA and HtrB, which degrade misfolded proteins [8], [9], [10]. It has been described that although *cssS* or *cssR*-null mutants are viable, the high level of alpha-amylase (AmyQ) secretion results in a significant reduction in the growth rate of the mutant strains [8]; on the other hand, mutations in either *htrA* or *htrB* do not have a noticeable effect on growth or secretory protein yield [11], [12]. However, the absence of HtrA leads to an increased synthesis of HtrB and vice versa [11].

In order to adapt to their natural environment, largely formed of insoluble polymers, Streptomycetes produce and secrete large quantities of suitable proteins [13], such as the more prevalent hydrolytic enzymes, together with antibiotics and signalling molecules. *Streptomyces lividans*, in particular, has often been used as a host for the secretory production of homologous and heterologous proteins [13], [14], [15], [16], [17], [18], but its secretion stress response still remains to be characterised. Misfolded secretory proteins could accumulate outside the cytoplasm, potentially interfering with essential bacterial cell processes, hence, the identification and characterisation of quality control factors involved in the elimination of misfolded proteins are of extraordinary importance to optimize secretory protein production in bacteria.

Hereby we report the identification of a novel CssRS-like two-component system governing the expression of three HtrA-like proteases, HtrB, HtrA1 and HtrA2 that seem to form part of the secretion stress response in *Streptomyces lividans* when alpha-amylase is oversynthesised.

## Results

### Identification of the *S. lividans* secretion stress response components

Based on the complete genome sequence of *Streptomyces coelicolor* A3(2) [19], a known closely-related strain to *Streptomyces lividans*, a search for the *S. lividans* homologue to the two-component system of the *B. subtilis* CssRS was performed. From the several potential equivalent systems found, the SCO4156-SCO4155 proteins, showing 34% and 21% identical residues in stretches of 170 and 301 residues respectively to the corresponding *B. subtilis* counterparts, were selected, due to them being the only two-component system with a gene encoding a HtrA-like protease (SCO4157) adjacent to it, a similar chromosomal organisation to that of *B. subtilis*. SCO4156-SCO4155 seems to form a unique transcriptional unit where the stop codon of the potential SCO4156 transcript overlaps with the start codon of the SCO4155 transcript, a situation widely accepted as a strong suggestion of both genes forming a single transcriptional unit, as is the case with the equivalent *B. subtilis* and *E. coli* genes. SCO4157 shares 32% identity with the HtrB protease of *B. subtilis* in a stretch of 271 residues. In a further search for the best homologues to the *B. subtilis* HtrA-like protease three additional candidates were identified: SCO2171, SCO3977 and SCO5149, with 35%, 35% and 36% identity in stretches of 288, 297 and 284 residues, respectively. A similar degree of identity was obtained in all cases when the deduced amino acid sequences of the different proteins were compared to the *E. coli* equivalents. Thus, the two component system SCO4156-SCO4155 was named CssRS and proteases SCO4157, SCO2171, SCO5149, SCO3977, were named HtrB, HtrA1, HtrA2 and HtrA3, respectively, and henceforth will be referred to as such.

### CssS or CssR deficiencies affect the transcriptional levels of the genes encoding the HtrA-like proteins

*S. lividans* Δ*cssS* and *S. lividans* Δ*cssR* mutant strains having disrupted *cssS* or *cssR* genes respectively were constructed, as indicated in Materials and Methods.

**Figure 1:**
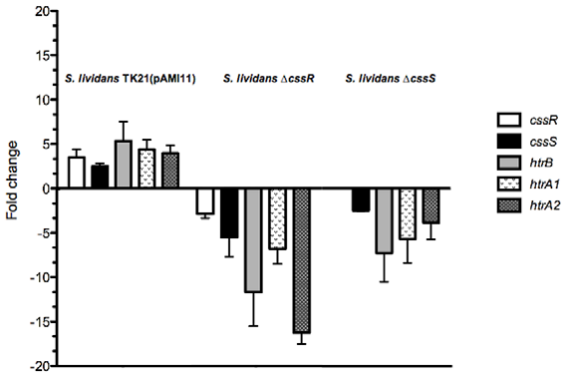
Regulation of the *S. lividans* secretion stress response. Quantitative RT-PCR analyses showed that the expression of the *cssS*, *cssR* and the three HtrA-like protease genes was upregulated in the *S. lividans* wild type strain overproducing *amlB*, *S. lividans* TK21 (pAMI1), whereas their expression resulted down regulated in the *S.lividans* Δ*cssR* and *S. lividans* Δ*cssS* strains. The expression of *cssR* was not affected in the CssS mutant strain. The values are the mean of at least three biological replicates. Bars show standard error.

To determine if the expression of the genes encoding the identified HtrA-like proteases were under the control of the two-component genes operon, the synthesis of the respective mRNAs from all the genes involved was analysed by quantitative RT-PCR (qRT-PCR) at 24 hours of growth. The expression of *cssR* or *cssS* was down regulated in their respective mutant strains, as expected. Expression of *htrB*, *htrA1* and *htrA2* was also down regulated in each of the two mutant strains (Fig. 1), strongly suggesting that the operon encoding the two-component genes was responsible for regulating the expression of the different protease genes. The expression of the third potential HtrA-like gene (SCO3977) resulted unaffected in any of the mutant strains and hence the gene was no longer considered as forming part of the set of genes being modulated by the two genes operon. Additionally, the expression of the *cssS* gene was also down regulated in the CssR deficient strain, whereas the expression of *cssR* remained unaltered in the CssS deficient strain, thus suggesting the existence of a polar effect of the first gene of the operon in the transcriptional level of the downstream gene. The expression levels for the *B. subtilis cssRS* equivalent operon has been reported to be naturally low [9].

### Alpha-amylase overproduction induces the expression of the *cssRS* operon

High-level production of alpha-amylase (AmyQ) of *Bacillus amyloliquefacies* activates the CssRS two-component system in *B. subtilis* [8]. To determine if CssS, CssR and the three HtrA-like proteases were involved in the equivalent secretion stress response in *Streptomyces lividans*, the pAMI11 plasmid harbouring the *S. lividans amlB* gene under the control of its own promoter was propagated in the wild type and *cssS*, *cssR* mutant strains, to generate *S. lividans* TK21 (pAMI11), *S. lividans* Δ*cssS* (pAMI11) and *S. lividans* Δ*cssR* (pAMI11), respectively. The mutant strains did not seem to show differences in growth rate in liquid medium with that of the wild type strain, as described in *B. subtilis* [8]. Overexpression of *amlB* in the mutant strains seemed to cause some minor constraint in the growth rate of the mutant strains when compared to the wild type strain equally overexpressing *amlB*, which is far from what was reported for *B. subtilis*, and particularly in the case of the *B. subtilis* CssR deficient strain oversynthesising AmyQ [8].

The expression of *cssS*, *cssR* and the three HtrA-like protease genes was analysed by qRT-PCR in the *S. lividans* wild type and the CssR and CssS deficient strains overproducing AmlB. The results showed that all genes were induced at 24 h of growth (Fig. 1), whereas *amlB* overproduction in the mutant strains was unable to trigger induction of any of the genes (not shown), again confirming the regulatory role of the *cssRS* operon on the *S. lividans* secretion stress response.

AmlB is a secretory protein that is transported and secreted outside the *S. lividans* cell by the Sec pathway. Proteins secreted via the Sec route are translocated in a misfolded manner and need to be properly folded before attaining their functional state in the supernatant. Overproduction of misfolded proteins triggers the induction of the CssRS two-component system and synthesis of the specific set of HtrA-like proteases. Some specific proteins secreted outside the cell in a fully-folded state use a minor secretion route, the so-called Tat pathway. Therefore, when overproduced, these proteins are unlikely to induce the CssRS system, and the *S. coelicolor* agarase (DagA) has been shown to be one of the proteins able to use the Tat route [20]. When the *dagA* gene was propagated in multicopy in *S. lividans* (*S. lividans* (pAGAs5) strain), the relative level of expression of the *cssR*, *cssS* and the three HtrA-like encoding genes remained unaltered (not shown), thus confirming that the likely accumulation of a misfolded secretory protein of the Sec route, such as AmlB, was responsible for the induction of the *cssRS* two genes operon and, subsequently, that of the three genes encoding the HtrA-like proteases.

### The *cssS* and *cssR* mutant strains accumulate non-functional AmlB in the cell cultures supernatants

**Figure 2:**
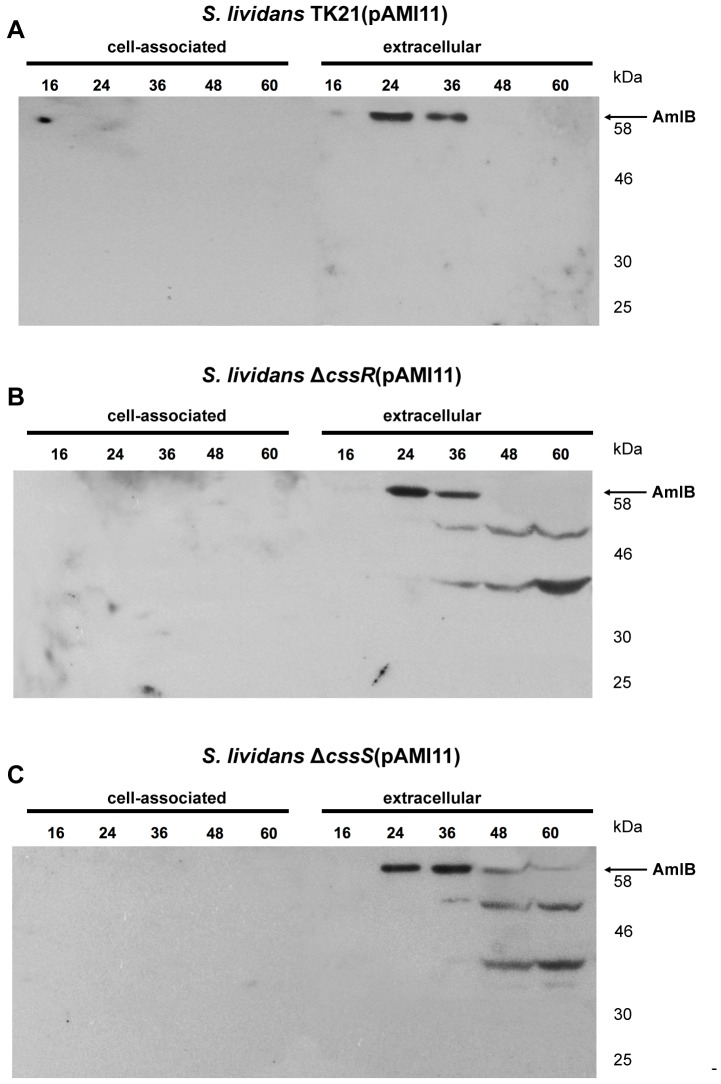
Alpha-amylase secretion pattern of *S. lividans* TK21 and the CssS and CssR deficient strains. Cell-associated and extracellular amylase present in *S. lividans* TK21 (pAMI11) (A), *S. lividans* Δ*cssR* (pAMI11) (B) and *S. lividans* Δ*cssS* (pAMI11) (C) at different times of growth were analyzed by Western blotting with antibodies raised against AmlB. The amount of protein loaded onto the gels was corrected by the dried weight of the bacterial cultures. Molecular size markers are indicated on the side of each panel. An arrow indicates the relative mobility of the mature AmlB.

The synthesis and secretion of the overproduced AmlB protein in the wild type and the CssR or CssS deficient strains were monitored by Western blot analysis. No precursor or mature forms of alpha-amylase were revealed by the anti-AmlB serum as associated to the cellular fraction of the wild type or the mutant strains, and AmlB was exclusively detected in the cultured cell supernatants in all cases (Fig. 2), strongly suggesting that synthesis, transport and secretion of the overproduced alpha-amylase took place very efficiently in all the strains. No α-amylase was detected in *S. lividans* TK21 (pIJ486) carried out as a control (not shown).

The observed molecular size (59 kDa) for the mature alpha-amylase was in accordance with that predicted from the derived amino acid sequence. The maximum level of mature AmlB appeared at the exponential phase of growth of the wild type bacterium, decreasing when the culture reached the stationary phase (Fig. 2A).

The maximum level of extracellular mature enzyme was mainly detected at the exponential phase of growth in the CssR- and CssS-deficient strains (Fig. 2B and C), although the pattern of degradation of the secreted alpha-amylase was different from that of the wild type strain since degradation occurred at a much slower pace. Basically, no net presence of mature protein was detected in the supernatants after the stationary phase of growth, and the degradation pattern of the secreted enzyme was very similar in both mutant strains (Fig. 2B and C).

**Figure 3:**
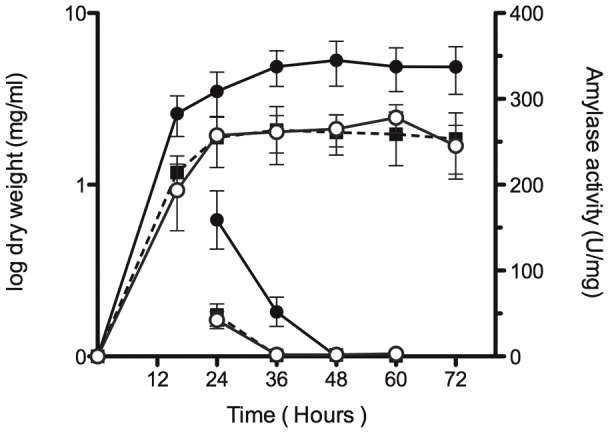
Alpha-amylase activity. Time-course of the *S. lividans* TK21 (pAMI11) (black circles), *S.lividans* Δ*cssR* (pAMI11) (white circles) and *S. lividans* Δ*cssS* (pAMI11) (black squares) bacterial cell cultures growing in NMMP medium (left Y axis). Alpha-amylase activity present in the culture supernatants was determined at the indicated times and is expressed as units per mg of protein (right Y axis). The data are the average of at least three independent determinations. Bars show standard error.

To investigate whether the alpha-amylase accumulated in the supernatant of both mutant strains was properly folded, the activity of the enzyme was measured and compared to that of the alpha-amylase present in the supernatant of the wild type strain. The alpha-amylase secreted in both mutant strains retained one third of the activity of that of the wild type strain at the exponential phase of growth, being inactive at later phases of growth (Fig. 3). The different degradation pattern of the alpha-amylase synthesised in the mutant strains and their considerably reduced level of enzymatic activity is consistent with the presence of extracellular misfolded non-active alpha-amylase in the supernatants of the mutant strains, and strongly suggests the involvement of the CssRS two-component system in the *S. lividans* secretion stress response.

## Discussion

The existence of quality systems to avoid the accumulation of misfolded proteins outside the cytoplasmic bacterial membrane is of paramount importance, since this protein accumulation is a major obstacle for the effective production of many secreted homologous or heterologous proteins in Gram-positive bacteria [3].

We have identified a two-component system and three proteases that appear to participate in the elimination of misfolded proteins in *Streptomyces lividans*, a well-known efficient secretory strain.

Among the 24 *Streptomyces* two-component systems that seem to share homology with the *Bacillus subtilis* CssRS proteins we selected three possible two-component systems as candidates, two of them encoding the two sensor proteins with the highest degree of homology (SCO5283-5282 and SCO2142-2143) and SCO4156-SCO4155, whose genes were the only ones close to a potential HtrA-like coding gene within a chromosomal organisation similar to that of the *Bacillus subtilis cssRS* two genes operon. The expression of the possible two-component systems was analysed in a strain of *S. lividans* oversynthesising alpha-amylase, *S. lividans* TK21(pAMI11). Only the SCO4156-SCO4155 operon was upregulated while the expression of the other two remained unaltered (data not shown), strongly suggesting that the upregulated operon was likely to be the one involved in the *S. lividans* secretion stress response. In fact, the transcriptional studies performed in the CssR deficient strain revealed the polar effect exerted on the *cssS* expression by the *cssR* mutation, thus suggesting the functionality of both genes as a single transcriptional unit, as is indeed the case in the *B. subtilis* and *E. coli* equivalent operons [21], [9].

The presence of an HtrA-like protease forming part of the secretion stress response has been found in several Gram-positive strains other than *B. subtilis* [22], [23], [24], [25].

Interestingly, an efficient secretory strain such as *B. subtilis* harbours two genes encoding HtrA-like proteins in its genome and both genes are modulated by a two–component system whose expression is triggered by the presence of secretory misfolded proteins [8], [9], [10]. The finding of up to three HtrA-like genes, whose expression is apparently controlled by the *S. lividans* two genes *cssRS* operon, although novel, may not be too surprising. In this regard, it is worth mentioning that overproduction of the DegU regulator in *S. coelicolor*, a closely related strain to *S. lividans*, does trigger overexpression of the major Sec route secretory proteins [26]. The relative expression levels of the *S. coelicolor* CssRS two-component system and the HtrA-like genes resulted also up regulated in the DegU overproducer strain (Table S1), thus enhancing the idea that the CssRS two-component system may be widely present and playing the same role in the *Streptomyces* strains.

Efficient secretory strains must govern their protein secretory systems very carefully, and the presence of the non-correctly folded proteins may interfere considerably with the secretion machinery exporting the mature secretory proteins outside the cell. Therefore, the bacterial cell must use efficient means to ensure that only properly folded proteins reach the extracellular space in a fully active conformation, where their activity needs to function to the maximum possible extent in the bacterial natural habitat. *B. subtilis* and *S. lividans* are soil bacteria adapted to live in their natural rough environments and must have evolved to develop that capacity to the full.

These proteases are induced in response to stresses that are likely to lead to protein misfolding. Bacterial secretory native proteins fold very rapidly into structures that are resistant to quality control proteases [3]. Therefore, it was not surprising to find that when the *S. lividans* native protein AmlB was oversynthesised, the mature protein appeared very rapidly in the cell culture supernatants (Fig. 2A), and that this phenotype was also observed when the enzyme was overproduced in the CssR- or CssS-deficient strains, however, in these cases the secreted protein was mainly carried to the cell culture supernatants in a misfolded state, although some correctly folded protein was also present outside the cell, which translates into a considerably reduced alpha-amylase activity (Fig. 3), as well as a different degree of access to the proteases regularly present in the cell culture supernatant (Fig. 2B and C). The correctly folded alpha-amylase made in the wild type strain is far more active as it has been properly folded during secretion.

Further research has been undertaken to better understand the regulatory events governing the action of the *S. lividans* CssRS two-component system on the expression of the HtrA-like protease genes. Experiments have been started to ascertain the relative role played by the different HtrA-like genes, and in particular, regarding the possible existence of any compensatory effects on the depletion of each protease gene or combinations of genes in a bacterium which, to our knowledge, is the first one reported to have three different HtrA-like protease genes involved in bacterial secretion stress response.

## Materials and Methods

### Bacterial strains, plasmids and media

The *S. lividans* TK21 [27] wild-type strain and its derivatives were cultured in liquid NMMP medium using mannitol as carbon source [27]. Apramycin (25 µg/ml), thiostrepton (50 µg/ml), kanamycin (50 µg/ml) and chloramphenicol (25 µg/ml) were added to the R5 and MS solid media, when required.

*S. lividans* Δ*cssS* and *S. lividans* Δ*cssR* are the *cssS* and *cssR* mutant strains where genes *cssS* and *cssR* have been disrupted. To construct the *cssS* mutant strain, oligonucleotides cssSdisrup_Fw (5′ GTTGGATCCGTGAACCGCTCCCTCAAC 3′) and cssSdisrup_Rv (5′GGTGAATTCTTGTCCAGGATGTTGACCAC 3′) were used to amplify a 627 nt DNA fragment from the *S. lividans* TK21 genome. To construct the *cssR* mutant strain, oligonucleotides cssRdisrup_Fw ( 5′GTTGGATCCACCTGGTCGTCCTGGACAT 3′) and cssRdisrup_Rv (5′GGTGAATTCACGGCCTTCAGGATCTGCT 3′) were used to amplify a 421 nt DNA fragment from the *S. lividans* TK21 genome. Both fragments were inserted into plasmid pOJ260 [28] through its unique *Bam*HI and *Eco*RI sites to generate plasmids pOJS and pOJR respectively, later used to conjugate *E. coli* to *Streptomyces*, as described [29]. *E. coli* ET12567 carrying the non-transmissible “driver” plasmid pUZ8002 was used for conjugation [30]. Apramycin resistant strains, *S. lividans* Δ*cssS* and *S. lividans* Δ*cssR*, containing the disrupted genes *cssS* and *cssR*, respectively, were selected upon verification of the disruption by PCR amplification and Southern blot hybridization analysis.

Plasmid pAMI11 [31] is a pIJ486 [32] derivative carrying the *S. lividans* gene *amlB* and a frame-shift-mutated thiostrepton resistance gene which was used to transform the *S. lividans* TK21, *S. lividans* Δ*cssS* and *S. lividans* Δ*cssR* protoplasts to generate *S. lividans* TK21(pAMI11), *S. lividans* Δ*cssS* (pAMI11) and *S. lividans*Δ*cssR* (pAMI11).

Plasmid pAGAs5 is a pAGAs1 [17] derivative containing the *S. coelicolor* agarase gene (*dagA*) and a frame-shift mutation in the thiostrepton resistance gene [33]. Plasmid pAGAs5 was used to transform *S. lividans* TK21 to obtain *S. lividans* TK21 (pAGAs5). Plasmid pIJ486 was propagated into the *S.lividans* TK21, *cssS* and *cssR* mutants to generate the corresponding isogenic strains.

### Quantitative real time PCR (qRT-PCR)

Total RNA was isolated from bacteria growing cultures using the RNeasy midi Kit (Qiagen). Cell lysates were extracted twice with phenol-chloroform before being loaded onto RNeasy midi columns for RNA purification. DNA potentially contaminating the RNA preparations was removed by incubation with RNase-free DNAse (Ambion) and its absence was tested by quantitative real time PCR amplification in the absence of reverse transcriptase. Complementary DNA was synthesised using the High Capacity Archive kit (Applied Biosystems). Quantitative real time PCR (qRT-PCR) was performed using SYBR Green technology in an ABI Prism 7300 Sequence Detection System (Applied Biosystems). Samples were initially denatured by heating at 95°C for 10 min. A 40-cycle amplification and quantification program was then followed (95°C for 15 sec and 60°C for 1 min) by a single fluorescence measurement per cycle, according to the manufacturer's recommendations. Subsequently a final extension cycle (72°C, 1 min) was performed. Three biological samples from the different bacterial cultures were amplified in triplicate in separate PCR reactions. All PCR products were between 50 and 150 bp in length.

A melting curve analysis was conducted after amplification to distinguish the targeted PCR products from the non-targeted ones. The melting curves were obtained by heating at temperatures ranging from 60°C to 95°C at a rate of 0.2°C per sec, with continuous fluorescence scanning. Oligonucleotides HRDBD (5′-GGACAAGCTGGCGAACTC -3′) and HRDBR (5′-CCTCCAGCAGGTGGTTCT -3′) were used to amplify the *hrdB* transcript carried out as an internal control to quantify the relative expression of the target genes. The *hrdB* transcript has currently been used as a reference to normalise the relative expression of *Streptomyces* genes [26], [34], [35], [36]. The oligonucleotides used as primers to amplify other transcripts are indicated in Table 1.

**Table 1:** Oligonucleotide primers used for gene transcript amplification.

Gene	Forward primer	Reverse primer
SLI2142	TGTTCCTGGAGCGGCAGTA	TCCCGCTGCTGGATGATCT
SLI2143	GACCTCGTCGTCCTGGACAT	GTGCGGTCAGCACGATCAC
SLI2171 (*htrA1*)	GGTGATCAAGGACGTACTG	GTCACCCGGAACGACTTC
SLI3471 (*dagA*)	GAAGGGGTATTTCGCAGATG	GGAATCCATTCCGAAGAAGA
SLI3977 (*htrA3*)	AGCAAGGCGTCCTACATGA	AGTTGATGCCGATGACGTT
SLI4155 (*cssS*)	TGGTCAACATCCTGGACAA	CCAGAAGCGGTCGAAGAC
SLI4156 (*cssR*)	CGAACTCCCTCGACGTGTA	CGGACCAGGCCTGTCACT
SLI4157 (*htrB*)	GAACGACGGCAAGCAGTA	GCGTTCTCCAGCTTGATCA
SLI5149 (*htrA*2)	ATCACCGTGACCTTCAACAG	TGACGCCCTTGACCTTCA
SLI5282	TGGACAACGGCGTCATAC	GACCATGTTGGCCTCCTT
SLI5283	AGGGATTCCTGGTGCAAAC	GCAGCATGATGTCGAGGAT
SLI7020 (*amlB*)	TGTTCGAGTGGAAGTTCACC	TCGACCATGCTCTTGAACTG

For clearer identification, the *S. coelicolor* gene nomenclature has been adopted for *S. lividans*, and the SCO acronym has been changed to SLI to indicate the strain of origin.

### Protein analysis and Western blot experiments

Standard extracellular protein analyses were essentially carried out as described [37]. Supernatants from cells grown in NMMP medium were collected by centrifugation at 1,400×g for 10 minutes. TCA was added at 10% final concentration to the supernatants and the mixture was incubated at −20°C for one hour to precipitate the extracellular proteins. The proteins were then separated by centrifugation at 15,000×g for 20 minutes at 4°C. Protein pellets were washed twice with ice-cold acetone and the residual acetone was removed by air-drying. Protein pellets were resuspended in 10 mM Tris-HCl (pH 7,5).

For intracellular protein analysis, the cell pellets were harvested by centrifugation at 1,400×g for 10 minutes, washed in P buffer [38], and sonicated on ice for three bursts of 5 s and re-suspended in P buffer containing the EDTA-free protease inhibitor cocktail (Roche).

For Western blot analysis, proteins were fractionated by SDS-PAGE in 10% (w/v) acrylamide gel [39] and transferred onto immobilon polyvinylidene difluoride membranes (Milipore), as described [40]. The protein concentration in the different samples was determined using the BCA protein assay kit (Pierce), as indicated by the supplier. The transferred material was incubated with rabbit polyclonal antibodies raised against *S. lividans* TK21 α-amylase (AmlB; a gift from C. Isiegas) followed by incubation with HRP-conjugated protein A (Invitrogen Laboratories) diluted 1∶10,000 in PBS containing 5% (w/v) skimmed milk for 40 min at room temperature [41]. Peptides reacting with the antibodies were revealed using the ECL enhanced chemiluminescence system from Amersham after a one min incubation and different exposures to X-ray film ranging from 20 s to 2 min.

### Enzyme activities

To determine extracellular alpha-amylase activity, the supernatants from 20-ml aliquots of bacterial cell cultures at the indicated phases of growth were concentrated by precipitation with ammonium sulphate brought to 80% saturation; the precipitated protein was collected by centrifugation at 13,000×g for 30 min and dissolved in 20 mM phosphate buffer (pH 7). The activity of alpha-amylase activity was estimated by determining the amount of reducing sugar released from starch. The assay was carried out by adding supernatant sample and starch solution 1% (w/v) treated with NaBH_4_, as described [42], in 20 mM phosphate buffer and was incubated at 37°C for 30 min. The reaction was stopped by the addition of dinitrosalicylic acid [43]. One unit of alpha-amylase was defined as the amount of enzyme necessary to produce reducing sugar equivalent to 1 µmol of glucose in 30 min under the assay conditions. The specific activity was expressed as units per mg of protein.

The protein concentration in the different samples was determined using the BCA protein assay kit (Pierce), as indicated by the supplier.

## Supporting Information

Table S1 **Quantitative RT-PCR analysis of the two-component system and the three genes encoding HtrA-like proteases in the** ***S. coelicolor*** **DegU overproducer strain.** Quantitative RT-PCR analysis of the two-component system and the HtrA- like protease genes in the *S. coelicolor* DegU overproducer strain (*S.coelicolor* M28; [26]) compared to that of the corresponding isogenic strain (*S. coelicolor* M145 [pIJ487]). The results correspond to the mean of at least three biological replicates, standard deviation are shown. *hrdB* was used as the reference gene to quantify the relative expression of the target genes. Oligonucleotide primers used to amplify the transcripts are indicated in Table 1.
(DOC)
