# Supplementary material for: A Novel Two-Component System Involved in Secretion Stress Response in Streptomyces lividans
Source: PLoS One. 2012 Nov 14;7(11):e48987. doi: 10.1371/journal.pone.0048987 (PMC3498368; doi:10.1371/journal.pone.0048987)
Supplement: Table S1 — Quantitative RT-PCR analysis of the two-component system and the three genes encoding HtrA-like proteases in the S. coelicolor DegU overproducer strain. Quantitative RT-PCR analysis of the two-component system and the HtrA- like protease genes in the S. coelicolor DegU overproducer strain (S.coelicolor M28; [26]) compared to that of the corresponding isogenic strain (S. coelicolor M145 [pIJ487]). The results correspond to the mean of at least three biological replicates, standard deviation are shown. hrdB was used as the reference gene to quantify the relative expression of the target genes. Oligonucleotide primers used to amplify the transcripts are indicated in Table 1. (DOC) [file pone.0048987.s001.doc]

**Table S1.** **Quantitative RT-PCR analysis of the two-component system and the three genes encoding HtrA-like proteases in the *S. coelicolor* DegU overproducer strain.**

| **Gene** | **Ratio M28 / M145 [pIJ487]** |
| --- | --- |
| *cssR* | 3,20 ± 0,75 |
| *cssS* | 2,17 ± 0,62 |
| *htrB* | 10,41± 0,71 |
| *htrA1* | 4,02 ± 0,54 |
| *htrA2* | 10,12 ± 0,76 |

Quantitative RT-PCR analysis of the two-component system and the HtrA- like proteases genes in the *S. coelicolor* DegU overproducer strain ( *S.coelicolor* M28;

[26] ) compared to that of the corresponding isogenic strain (*S. coelicolor* M145 [pIJ487]). The results correspond to the mean of at least three biological replicates, standard deviation are shown. *hrdB* was used as the reference gene to quantify the relative expression of the target genes. Oligonucleotide primers used to amplify the transcripts are indicated in Table 1.
